# Re-exploration of prognosis in type B thymomas: establishment of a predictive nomogram model

**DOI:** 10.1186/s12957-023-03293-2

**Published:** 2024-01-23

**Authors:** Ke Zhao, Lei Liu, Xiaoyun Zhou, Guige Wang, Jiaqi Zhang, Xuehan Gao, Libing Yang, Ke Rao, Chao Guo, Ye Zhang, Cheng Huang, Hongsheng Liu, Shanqing Li, Yeye Chen

**Affiliations:** 1 Department of Thoracic Surgery, Peking Union Medical College Hospital, Chinese Academy of Medical Science & Peking Union Medical College, No.1 Shuaifuyuan, Dongcheng District, Beijing, 100730 China; 2 Peking Union Medical College Hospital, Chinese Academy of Medical Science & Peking Union Medical College, Beijing, China

**Keywords:** Nomogram, Thymoma, Predictive model, Type B, Prognosis

## Abstract

**Objective:**

To explore the risk factors for disease progression after initial treatment of type B thymomas using a predictive nomogram model.

**Methods:**

A single-center retrospective study of patients with type B thymoma was performed. The Cox proportional hazard model was used for univariate and multivariate analyses. Variables with statistical and clinical significance in the multivariate Cox regression were integrated into a nomogram to establish a predictive model for disease progression.

**Results:**

A total of 353 cases with type B thymoma were retrieved between January 2012 and December 2021. The median follow-up was 58 months (range: 1–128 months). The 10-year progression-free survival (PFS) was 91.8%. The final nomogram model included R0 resection status and Masaoka stage, with a concordance index of 0.880. Non-R0 resection and advanced Masaoka stage were negative prognostic factors for disease progression (*p* < 0.001). No benefits of postoperative radiotherapy (PORT) were observed in patients with advanced stage and non-R0 resection (*p* = 0.114 and 0.284, respectively).

**Conclusion:**

The best treatment strategy for type B thymoma is the detection and achievement of R0 resection as early as possible. Long-term follow-up is necessary, especially for patients with advanced Masaoka stage and who have not achieved R0 resection. No prognostic benefits were observed for PORT.

## Introduction

Thymoma is a malignant tumor originating from thymic epithelial cells and is the most common primary neoplasm of the anterior mediastinum [1]. According to the latest histological classification of the World Health Organization (WHO), thymoma can be divided into five main histological subtypes (A, AB, B1, B2, and B3), among which B1, B2, and B3 are collectively referred to as type B thymoma [2, 3]. These three subtypes can coexist in any proportion in the same thymoma. This condition was previously defined as combined thymoma. However, the International Thymic Malignancy Interest Group (ITMIG) recommended the abandonment of this name and proposed listing all tumor subtypes in order of dominance during diagnosis [3, 4]. While the prognosis of type B thymoma has been previously reported, data on prognosis-related factors remain controversial [5–7].

A nomogram is a visual statistical model widely used for predicting the prognosis of various cancers [8–10]. It does not require the categorization of continuous variables and transforms complex regression equations into intuitive graphs [11]. A nomogram is easy to use, especially when only a small number of predictor variables are included in the model, making the assessment of patient prognosis more convenient [11]. Therefore, the present study proposes a new nomogram model to predict progression-free survival (PFS) in patients with type B thymoma.

## Methods

### Ethical statement

This study was approved by the Institutional Review Board (IRB) of Peking Union Medical College Hospital (PUMCH; approval number: K4672). Informed consent was waived by the IRB.

### Patient selection

Data from all thymoma patients at PUMCH were retrospectively collected from January 2012 to December 2021. Patients received initial treatment at PUMCH, with surgery, radiotherapy, chemotherapy, interventional ablation, or immunotherapy as treatment options. Five patients who did not receive any treatment were excluded. Patients with type A thymoma (45 cases), type AB thymoma (168 cases), and other special types of thymoma (23 cases) were excluded. One patient was excluded due to perioperative death. Finally, 353 patients with B-type thymoma were retrospectively analyzed, including patients with single and multiple pathological subtypes.

### Histology and staging

All patient diagnoses were confirmed by surgical or biopsy specimens and were reviewed by experienced pathologists. The stage of thymoma was determined according to the modified Masaoka staging system [12]. The modified Masaoka stage of the patient was jointly determined by a thoracic surgeon and pathologist.

### Follow-up

The primary endpoint was disease progression. Local recurrence and distant metastasis were both considered as disease progression, which was estimated through chest computed tomography (CT) or positron emission tomography/CT (PET/CT). The duration of PFS was calculated from the date of initial treatment until the last follow-up or the detection of disease progression. Follow-up information was obtained through outpatient services or phone calls.

### Statistical analysis

Descriptive statistics were expressed as the median, quartile, range, mean, and standard deviation for continuous variables and the frequency and proportion for categorical variables. The *t*-test was used to compare differences between the mean values for continuous variables, while the chi-square test and Fisher’s exact test were used to compare differences between the proportions for categorical variables. Cox regression models were used for univariate and multivariate analyses and estimated using hazard ratios (HR) and 95% confidence intervals (CI) statistics. The performance of the prediction models was assessed and compared using the concordance index (c-index) [13]. *p*-values less than 0.05 were considered statistically significant. R 4.2.3 was used for statistical analysis and plotting.

## Results

### Patient characteristics

A total of 353 patients were enrolled, of which 107 patients had type B1 thymoma, 134 had type B2 thymoma, 63 had type B3 thymoma, and 49 had type B thymoma with coexistence of multiple histological subtypes. Baseline clinicopathological characteristics are in Table 1. Patients were grouped based on the presence or absence of disease progression. In addition, 6 patients suffered from non-myasthenia gravis paraneoplastic diseases, including 2 cases of Sjogren’s syndrome, 1 case of primary thrombocytopenia, 1 case of Good’s syndrome, 1 case of psoriasis, and 1 case of autoimmune encephalitis.

**Table 1:** Baseline characteristics of patients with type B thymomas

Variables	Overall	No progression	Progression	*p*-value
**Size (mm, median, quartile, md = 5)**	48.50 (35.00, 62.75)	45.00 (32.00, 60.00)	65.00 (50.00, 85.00)	** < 0.001**
**Age (years, median, quartile, md = 0)**	49.00 (40.00, 58.00)	50.00 (40.00, 58.25)	44.00 (36.00, 51.00)	**0.026**
**Pathologic type (%, md = 0)**				0.342
** Type B1**	107 (30.3)	89 (29.7)	4 (13.8)	
** Type B2**	134 (38.0)	114 (38.0)	13 (44.8)	
** Type B3**	63 (17.8)	55 (18.3)	7 (24.1)	
** Type B thymoma with multiple histological subtypes coexisting**	49 (13.9)	42 (14.0)	5 (17.2)	
**Smoking (%, md = 0)**				0.267
** No**	269 (76.2)	230 (76.7)	19 (65.5)	
** Yes**	84 (23.8)	70 (23.3)	10 (34.5)	
**Masaoka stage (%, md = 0)**				** < 0.001**
** Early stage**	246 (69.7)	227 (75.7)	2 (6.9)	
** Advanced stage**	107 (30.3)	73 (24.3)	27 (93.1)	
**Gender (%, md = 0)**				0.626
** Female**	167 (47.3)	144 (48.0)	12 (41.4)	
** Male**	186 (52.7)	156 (52.0)	17 (58.6)	
**Myasthenia gravis (%, md = 0)**				0.794
** No**	201 (56.9)	173 (57.7)	18 (62.1)	
** Yes**	152 (43.1)	127 (42.3)	11 (37.9)	
**Surgery approach (%, md = 10)**				** < 0.001**
** Minimal invasive**	231 (67.3)	214 (71.6)	5 (23.8)	
** Open**	112 (32.7)	85 (28.4)	16 (76.2)	
**R0 resection status (%, md = 2)**				** < 0.001**
** Yes**	329 (93.7)	292 (97.3)	14 (51.9)	
** No**	22 (6.3)	8 (2.7)	13 (48.1)	
**Radiotherapy (%, md = 22)**				**0.001**
** No**	183 (55.3)	173 (58.2)	7 (24.1)	
** Yes**	148 (44.7)	124 (41.8)	22 (75.9)	
**Chemotherapy (%, md = 22)**				** < 0.001**
** No**	290 (87.6)	268 (90.2)	18 (62.1)	
** Yes**	41 (12.4)	29 (9.8)	11 (37.9)	

*Abbreviation*: *md* missing data

### Follow-up and outcome

The median follow-up time was 58 months (range: 1–128 months). Twenty-eight patients were lost to follow-up and 24 patients were unable to obtain information on disease progression. Eight patients passed away, four of which died of thymoma recurrence, or metastasis, and the remaining ones died of unknown causes.

### Disease progression prediction and prognostic factors by nomogram

During the follow-up period, 29 patients experienced disease progression, with a median progression time of 32 months (range: 6–105 months). Most of these patients exhibited intrathoracic metastasis (22/29, 75.9%), 4 patients had local recurrence, 2 patients had distant metastasis, and 1 patient had an unknown progression site.

Univariate analysis was used to evaluate the effects of gender, age, Masaoka stage, R0 resection status, size, radiotherapy, chemotherapy, myasthenia gravis (MG), smoking, surgical approach, and tissue type on PFS (Table 2). All statistically significant factors in univariate analysis were included in multivariate analysis (Table 2). The results showed that R0 resection status, Masaoka stage, and radiotherapy were independent prognostic factors for disease progression. Non-R0 resection, advanced Masaoka stage, and radiotherapy were associated with an increased risk of disease progression, with an HR of 10.65 (95% CI: 3.10–36.51, *p* < 0.001), 11.33 (95% CI: 2.03–63.20, *p* = 0.006), and 4.02 (95% CI: 1.14–14.14, *p* = 0.030), respectively.

**Table 2:** Univariate and multivariate analyses of prognostic factors of PFS

Variables	Univariate analyses		Multivariate analyses	
	HR (95% CI)	*p* value	HR (95% CI)	*p* value
**Age**	0.97 (0.94–1.00)	**0.036**	0.99 (0.95–1.03)	0.465
**Surgery approach**	5.58 (2.04–15.30)	**0.001**	1.05 (0.29–3.75)	0.943
**Chemotherapy**	4.24 (2.00–8.98)	** < 0.001**	0.70 (0.17–2.88)	0.624
**Smoking**	1.61 (0.75–3.47)	0.221		
**Masaoka stage**	33.26 (7.91–139.89)	** < 0.001**	9.75 (1.97–48.22)	**0.005**
**Myasthenia gravis**	0.69 (0.33–1.47)	0.338		
**Pathologic type**	1.34 (0.94–1.90)	0.106		
**R0 resection status**	23.29 (10.69–50.76)	** < 0.001**	9.47 (3.16–28.44)	** < 0.001**
**Radiotherapy**	4.35 (1.86–10.21)	**0.001**	4.06 (1.15–14.42)	**0.030**
**Gender**	1.26 (0.60–2.63)	0.543		
**Size**	1.02 (1.01–1.03)	**0.001**	1.01 (0.99–1.03)	0.429

*Abbreviations*: *PFS* progression-free survival, *HR* hazard ratio, *CI* confidence interval

**Fig. 1:**
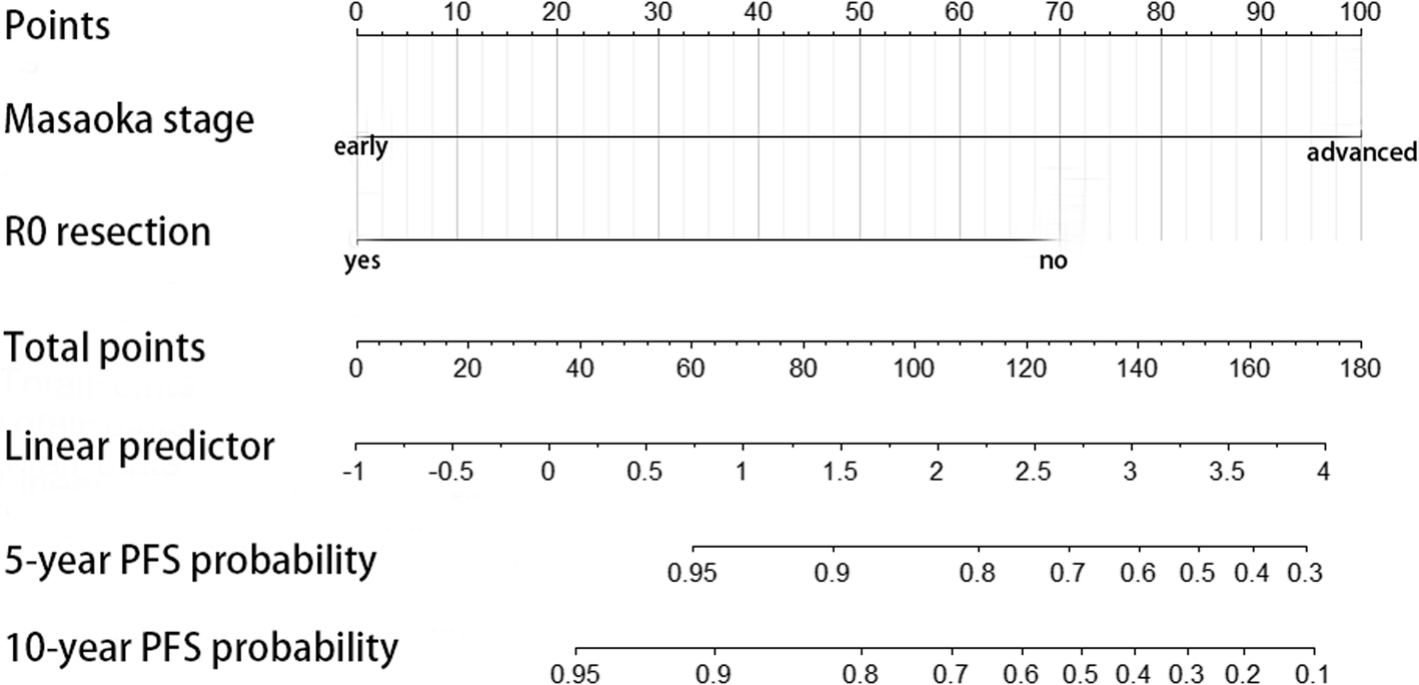
Nomogram for prediction of disease progression in patients with type B thymomas. PFS, progression-free survival

**Fig. 2:**
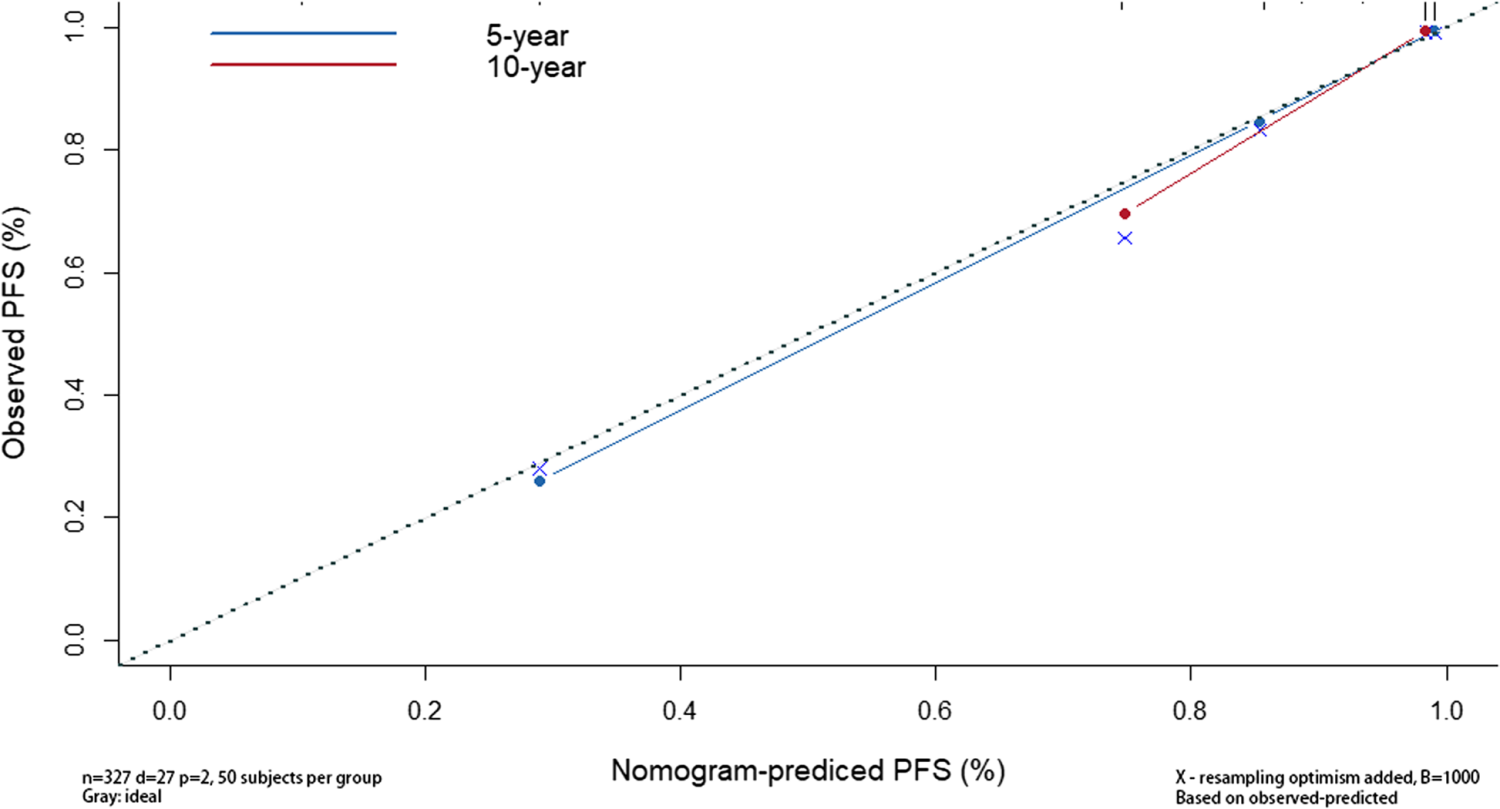
Calibration curves of the nomogram. The *x*-axis represents the nomogram-predicted probability and the *y*-axis represents the actual probability of PFS. Perfect prediction corresponds to the 45°green dashed line. PFS, progression-free survival

The final nomogram model included R0 resection status and Masaoka stage (Fig. 1). The nomogram showed that the Masaoka stage contributed the most, followed by R0 resection status. Each category of these variables was assigned a score. By adding up the total scores and positioning them on the total subscale, the probability of disease progression was easily estimated. The bootstrap-corrected C-index of this nomogram was 0.880 (95% CI: 0.852 to 0.909). The calibration curve showed good agreement between the predicted and actual PFS outcomes (Fig. 2).

## Discussion

To the best of our knowledge, this is the first nomogram model for the prediction of PFS in patients with type B thymoma and the largest single-center retrospective study of type B thymoma in the Chinese population. Multivariate analysis showed that non-R0 resection, advanced Masaoka stage, and radiotherapy were independent risk factors for the disease progression in patients with type B thymoma. In addition, we have also provided negative evidence for some controversial issues, such as the significance of postoperative radiotherapy and pathological types on prognosis of type B thymoma. We believe that summarizing diagnosis and treatment experience as much as possible for this rare disease is beneficial for clinical work.

It has been previously suggested that R0 resection is an important factor in predicting the disease progression in patients with thymoma [14–18], corresponding to our finding. Both univariate and multivariate analyses indicated that patients with R0 resection had a lower proportion of disease progression compared with those without R0 resection (*p* < 0.001, Tables 1 and 2). Non-R0 resection patients often receive other treatments. The present study found that 22 patients were unable to achieve R0 resection, of which 18 patients received comprehensive treatment, including radiotherapy or chemotherapy. However, 61.9% of patients still experienced disease progression. The impact of surgical and chemoradiotherapy sequences on prognosis was not compared owing to the small number of cases. However, according to previous studies, preoperative chemotherapy can increase the likelihood of R0 resection, thereby improving patient prognosis [17, 19].

Modified Masaoka staging is an independent risk factor for the prognosis of all types of thymoma, whether with overall survival or PFS as the endpoint [7, 18, 20, 21]. This conclusion has also been confirmed in multiple studies targeting type B thymoma [5, 14, 17, 22]. Similarly, the Masaoka stage was also an important disease prognostic factor for disease progression in our cohort. Given the small number of stage IV patients (22 cases), stage III and IV patients were classified as advanced-stage patients and analyzed together. This group exhibited a great heterogeneity, further analysis revealed that the prognosis of stage IV patients was significantly worse than that of stage III patients (*p* < 0.001). In early-stage patients, only 2 cases of disease progression occurred in stage IIB patients, whereas 196 patients in stage I had no disease progression. Tseng et al. [23] analyzed the impact of different metastasis locations on prognosis in Masaoka stage III patients and found that invasion of great vessels such as the innominate vein or superior vena cava was associated with a higher risk of disease recurrence. However, there was no statistical difference in the proportion of disease progression between the invasion of great vessels (5/19) and other locations (9/66) (*p* = 0.189).

The results of multivariate Cox analysis showed that postoperative radiotherapy (PORT) was a poor prognostic factor for the disease progression in patients with type B thymoma, which is counterintuitive. Jackson et al. [24] conducted a large-scale retrospective study on 4056 patients with thymoma and found that PORT was associated with longer overall survival, with the greatest relative benefit observed in patients with Masaoka stage IIB-III diseases and non-R0 resection. Similarly, Lim et al. [25] reported that PORT may benefit patients in Masaoka stage III-IV. Some studies also pointed out that PORT is not an independent prognostic factor for patients with type B thymoma [5, 17]. However, no study has demonstrated that PORT can lead to poor prognosis in patients with thymoma. Therefore, after reanalysis of data, we believed this may be because the good prognosis of early-stage patients exaggerates the significance of non-radiation therapy for patients with type B thymoma. We found that the proportion of early Masaoka patients receiving radiotherapy was much lower than that of advanced-stage patients (*p* < 0.001). It was also found that the disease progression rate of early-stage patients was significantly lower than that of advanced-stage patients (*p* < 0.001, Table 1). And we found no benefits of PORT even in patients with advanced stage and non-R0 resection (*p* = 0.114 and 0.284, respectively).

A previous study found that histological types B2 and B3 were independent risk factors for poor prognosis compared with type B1 thymoma [26], it is believed that there are significantly more Masaoka stage I and II cases of B1 type tumors than B2 and B3 type tumors, and their complete resection rate is also significantly higher than the latter, which may lead to better prognosis. Patients in the present study also exhibited this characteristic; the proportion of early Masaoka stage and R0 resection patients was higher (86.9% vs 62.2%, *p* < 0.001; 98.1% vs 91.1%, *p* = 0.015). Nonetheless, Wright et al. [27] inferred that the prognosis of B1 and B2 thymoma is similar, while the prognosis of B3 thymoma patients is even worse. Other studies found no difference in the recurrence rate among different subtype B thymomas [5, 7], which is consistent with our results, although the disease progression rate of type B1 was lower than that of other subtypes (*p* = 0.070).

Therefore, our final nomogram model only included two elements: R0 resection and Masaoka staging, and the predictive ability of the model was tested through validation curves. The calibration curves show that when the predicted disease progression probability was low (below 95%), the 5- and 10-year disease progression probabilities were slightly overestimated, and after exceeding 95%, the predicted probability was very close to the actual situation (Figs. 1 and 2).

The 5- and 10-year cumulative mortality rate for all patients was 1.4% and 2.3%, respectively, and the 5- and 10-year cumulative disease progression rate (CDPR) was 3.1% and 8.2%, respectively. Furthermore, the patterns of disease progression were analyzed, and it was found that 89.7% of disease progression was limited to the chest and mediastinum, and 86.2% of disease progression occurred within 5 years after initial treatment. Based on these findings, we recommended the following follow-up strategies for patients with type B thymoma. R0 resection and early Masaoka stage patients should undergo chest CT examination every 12 months for 10 years. If both conditions are not met simultaneously, then patients should undergo a chest CT examination every 6 months for 5 years, followed by a chest CT examination every 12 months for the subsequent 5 years. However, routine screening for systemic metastases is not recommended.

Nonetheless, this study still has some limitations. Firstly, it is limited by its retrospective design, and some data are missing, such as some patients did not undergo lymph node resection during surgery, which leads to the inability to analyze the TNM stage of the patients. Secondly, there is a certain degree of hospitalization bias in this study, the number of advanced Masaoka stage patients enrolled, especially those in stage IV, is relatively small, which may lead to excessive positive estimates of the prognosis of B-type thymoma. Finally, as this is a single-center study, the promotion of conclusions should be carefully considered.

## Data Availability

The data underlying this article comes from PUMCH, and this study was approved by the IRB of PUMCH (approval number: K4672). Informed consent was waived by the IRB. The data underlying this article will be shared on reasonable request to the corresponding author.
